# Myogenic Anti-Nucleolin Aptamer iSN04 Inhibits Proliferation and Promotes Differentiation of Vascular Smooth Muscle Cells

**DOI:** 10.3390/biom14060709

**Published:** 2024-06-15

**Authors:** Mana Miyoshi, Takeshi Shimosato, Tomohide Takaya

**Affiliations:** 1 Department of Agriculture, Graduate School of Science and Technology, Shinshu University, 8304 Minami-minowa, Kami-ina, Nagano 399-4598, Japan; 2 Department of Agricultural and Life Sciences, Faculty of Agriculture, Shinshu University, 8304 Minami-minowa, Kami-ina, Nagano 399-4598, Japan; 3 Department of Biomolecular Innovation, Institute for Biomedical Sciences, Shinshu University, 8304 Minami-minowa, Kami-ina, Nagano 399-4598, Japan

**Keywords:** aptamer, myogenetic oligodeoxynucleotide, nucleolin, vascular smooth muscle cell

## Abstract

De-differentiation and subsequent increased proliferation and inflammation of vascular smooth muscle cells (VSMCs) is one of the mechanisms of atherogenesis. Maintaining VSMCs in a contractile differentiated state is therefore a promising therapeutic strategy for atherosclerosis. We have reported the 18-base myogenetic oligodeoxynucleotide, iSN04, which serves as an anti-nucleolin aptamer and promotes skeletal and myocardial differentiation. The present study investigated the effect of iSN04 on VSMCs because nucleolin has been reported to contribute to VSMC de-differentiation under pathophysiological conditions. Nucleolin is localized in the nucleoplasm and nucleoli of both rat and human VSMCs. iSN04 without a carrier was spontaneously incorporated into VSMCs, indicating that iSN04 would serve as an anti-nucleolin aptamer. iSN04 treatment decreased the ratio of 5-ethynyl-2′-deoxyuridine (EdU)-positive proliferating VSMCs and increased the expression of α-smooth muscle actin, a contractile marker of VSMCs. iSN04 also suppressed angiogenesis of mouse aortic rings ex vivo, which is a model of pathological angiogenesis involved in plaque formation, growth, and rupture. These results demonstrate that antagonizing nucleolin with iSN04 preserves VSMC differentiation, providing a nucleic acid drug candidate for the treatment of vascular disease.

## 1. Introduction

Atherosclerosis is a major risk factor for cardiovascular disease, the leading cause of death worldwide [1]. Vascular smooth muscle cells (VSMCs) in the arterial media play a key role in the development and rupture of atherosclerotic plaques [2]. Unlike skeletal and cardiac muscle cells, smooth muscle cells (SMCs), including VSMCs, can plastically switch their phenotypes of proliferation and differentiation. In the normal state, differentiated VSMCs express SMC markers such as α-smooth muscle actin (α-SMA), transgelin (SM22α), and caldesmon to maintain their contractile properties required for blood vessels [3]. However, during atherogenesis, VSMCs, macrophages, and their extracellular matrix form the primary core of the plaque in the media. In particular, apoptotic macrophages in the plaque produce growth factors, cytokines, and matrix metalloproteinases that switch the VSMC phenotype [4]. De-differentiated VSMCs reduce the levels of SMC markers, increase their proliferative capacity, and migrate from the media to the intima. The migrated VSMCs form the fibrous cap that appears to stabilize the plaque, but lipid accumulation transforms the VSMCs into foam cells and leads to apoptosis, resulting in plaque rupture and ultimately myocardial infarction or stroke [2,4]. Not only lipids but also oxidative stress in the hypoxic regions within the developed plaque promotes microvessel formation [5]. Because such intraplaque angiogenesis destabilizes the plaque, pharmacological inhibition of neovascularization associated with VSMC recruitment has been studied [5,6,7]. Overall, the regulation of VSMC de-differentiation or phenotype switching is an important issue in vascular therapy. It should be noted that de-differentiation and proliferation of VSMCs occur not only in atherosclerosis but also in restenosis, calcification, and pathological angiogenesis [8]. Maintaining VSMCs in a contractile differentiated state is an important strategy for the prevention and therapy of vascular disease.

We have reported a series of myogenetic oligodeoxynucleotides (myoDNs), which are 12–18-base single-stranded oligodeoxynucleotides that promote myogenesis [9,10]. One of the myoDNs, iSN04 (5′-AGA TTA GGG TGA GGG TGA-3′), serves as an anti-nucleolin aptamer and induces myogenic differentiation of myoblasts [9,11] and rhabdomyosarcoma cells [12]. Since iSN04 could express myogenic action even in diabetic and cachectic conditions [13,14], it is expected to be a drug seed for muscle wasting in chronic disease patients. iSN04 physically interacts with nucleolin, a multifunctional phosphoprotein that is ubiquitously expressed and changes its subcellular localization depending on biological processes such as proliferation, differentiation, and inflammation [15]. In myoblasts, nucleolin binds to p53 mRNA to interrupt its translation, but antagonizing nucleolin with iSN04 releases p53 mRNA from nucleolin and improves p53 protein levels, resulting in enhanced myogenesis [9]. Interestingly, iSN04 facilitates myocardial differentiation of pluripotent stem cells by modulating the Wnt signaling pathway [16], suggesting that nucleolin antagonism by iSN04 affects not only skeletal muscle but also multiple muscle lineages. Indeed, iSN04 exerted anti-inflammatory effects on both skeletal and smooth muscle cells by suppressing the β-catenin/nuclear factor-κB (NF-κB) signaling pathway [17]. These studies demonstrate that iSN04 is a potential nucleic acid drug for skeletal, cardiac, and smooth muscle dysfunction, but its action on VSMC differentiation has not been investigated.

Nucleolin, the target of iSN04, has been reported to localize to the nucleoli of rat aortic SMCs [18]. In VSMCs, angiotensin II induces nucleolin expression and its translocation from the nucleus to the cytoplasm. Cytoplasmic nucleolin binds and stabilizes epidermal growth factor (EGF) and platelet-derived growth factor (PDGF) mRNAs, leading to a phenotypic transformation that decreases SMC markers and increases proliferation [19,20]. In *ApoE*^-/-^ mice fed a high-fat diet, nucleolin expression was upregulated in VSMCs within advanced aortic plaques. Oxidized low-density lipoprotein (oxLDL), an atherogenic cholesterol, increases nucleolin, which is predicted to interact with aurora B to promote the cell cycle [21]. Nucleolin is also involved in oxLDL-induced foam cell formation [22]. In pulmonary arterial SMCs, hypoxia induces nucleolin translocation from the nucleus to the plasma membrane. Neurite growth-promoting factor 2 (midkine) interacts with cell surface nucleolin and activates EGF receptor signaling to promote migration and proliferation [23]. These studies show that nucleolin contributes to the de-differentiation and proliferation of VSMCs under pathophysiological conditions and strongly suggest that antagonizing nucleolin would be beneficial for VSMC treatment.

The present study investigated the effect of the myogenetic anti-nucleolin aptamer, iSN04, on the proliferation and differentiation of VSMCs in vitro and on the angiogenesis of aortic rings ex vivo. As expected, iSN04 maintained VSMC differentiation, providing a feasible scheme for atherosclerosis therapy.

## 2. Materials and Methods

### 2.1. Chemicals

iSN04, in which all phosphodiester bonds were phosphorothioated to enhance nuclease resistance in cell culture, was synthesized and HPLC-purified (GeneDesign, Osaka, Japan). iSN04 was dissolved in endotoxin-free water, and an equal volume of water without iSN04 was used as a negative control. 6-FAM-iSN04 is the iSN04 conjugated to 6-carboxyfluorecein at the 5′ end (GeneDesign) [9].

### 2.2. Cell Culture

All cells were cultured at 37 °C with 5% CO_2_ throughout the experiments.

Embryonic rat thoracic aortic SMC line A10 (CRL-1476; ATCC, Manassas, VA, USA) was maintained in growth medium (GM) consisting of DMEM (Nacalai, Osaka, Japan), 10% fetal bovine serum (FBS) (GE Healthcare, Chicago, IL, USA), and a mixture of 100 units/mL penicillin and 100 μg/mL streptomycin (P/S) (Nacalai). A10 cells were induced to differentiate in differentiation medium (DM) consisting of DMEM, 2% horse serum (HyClone; Cytiva, Marlborough, MA, USA), and P/S.

The commercially available human aortic SMC (hAoSMC) stocks from a healthy 55-year-old male and a 51-year-old female (C-12533, lot 437Z012.2 and 437Z016.2, respectively; PromoCell, Heidelberg, Germany) were maintained using the Smooth Muscle Cell Growth Medium 2 Kit (C-22162; PromoCell) as GM for hAoSMC (hGM) and induced to differentiate in DM for hAoSMC (hDM), consisting of DMEM, 1% FBS, and P/S.

### 2.3. Immunocytochemistry

A total of 1.0 × 10^5^ A10 cells and 7.5 × 10^4^ hAoSMCs were seeded on 30 mm dishes. For α-SMA staining, A10 cells were treated the next day with 3 or 10 μM iSN04 in DM for 4 days. The cells were fixed with 2% paraformaldehyde, permeabilized with 0.2% Triton X-100, and immunostained with 1.0 μg/mL rabbit polyclonal anti-nucleolin antibody (ab22758; Abcam, Cambridge, UK) or 1.0 μg/mL mouse monoclonal anti-α-SMA antibody (1A4, ab7817; Abcam) overnight at 4 °C. The cells were then stained with 0.1 μg/mL Alexa Fluor 594- or 488-conjugated donkey polyclonal anti-rabbit or anti-mouse IgG antibody (Jackson ImmunoResearch, West Grove, PA, USA) for 1 h at room temperature. Cell nuclei were stained with 4′,6-diamidino-2-phenylindole (DAPI) (Nacalai). Fluorescence images were captured using an EVOS FL Auto microscope (AMAFD1000; Thermo Fisher Scientific, Waltham, MA, USA). SMA signal intensity was quantified using ImageJ software version 1.52a (National Institutes of Health, Bethesda, MD, USA).

### 2.4. Incorporation Assay

A total of 1.0 × 10^4^ A10 cells/well were seeded on 8-well glass chamber slides (SCS-N08; Matsunami Glass, Osaka, Japan) and treated the next day with 5 μg/mL 6-FAM-iSN04 in GM for 0.5, 1, 2, or 4 h in each well. The cells were then washed with PBS to remove free extracellular 6-FAM-iSN04, fixed with 2% paraformaldehyde, and stained with DAPI. Fluorescence images were captured using an EVOS FL Auto microscope with a ×40 objective magnification to visualize intracellularly incorporated 6-FAM-iSN04 [9].

### 2.5. 5-Ethynyl-2′-deoxyuridine (EdU) Staining

A total of 1.0 × 10^5^ A10 cells or 9.0 × 10^4^ hAoSMCs were seeded on 30 mm dishes and treated the next day with 30 μM iSN04 in GM for 24 h (A10) or 48 h (hAoSMC). EdU was administered at a final concentration of 10 μM, and the cells were cultured for 3 h (A10) or 12 h (hAoSMC). EdU staining was performed using the Click-iT EdU Imaging Kit (Thermo Fisher Scientific), according to the manufacturer’s instructions. Cell nuclei were stained with DAPI. Fluorescent images were captured using an EVOS FL Auto microscope. The ratio of EdU^+^ cells was defined as the number of EdU^+^ nuclei divided by the total number of nuclei using ImageJ software version 1.52a [12].

### 2.6. Quantitative Real-Time RT-PCR (qPCR)

A total of 1.0 × 10^5^ hAoSMCs were seeded on 60 mm dishes and treated the next day with 30 μM iSN04 in hGM for 48 h. Total RNA was isolated using NucleoSpin RNA Plus (Macherey-Nagel, Düren, Germany) and reverse transcribed using ReverTra Ace qPCR RT Master Mix (TOYOBO, Osaka, Japan). qPCR was performed using GoTaq qPCR Master Mix (Promega, Madison, WI, USA) with the StepOne Real-Time PCR System (Thermo Fisher Scientific). The amount of each transcript was normalized to that of 3-monooxygenase/tryptophan 5-monooxygenase activation protein zeta gene (*YWHAZ*), which was used as an internal control. The results are presented as fold changes. The primer sequences of α-SMA (*ACTA2*), caldesmon (*CALD1*), Ki-67 (*MKI67*), SM22α (*TAGLN*), and *YWHAZ* are listed in Table 1.

### 2.7. Aortic Ring Assay

Three-dimensional aortic ring angiogenesis was analyzed as previously reported [27]. Aortas from 9-week-old male C57BL/6J mice (Japan SLC, Shizuoka, Japan) were dissected, perfused with Opti-MEM (Thermo Fisher Scientific), cut into 1 mm rings, and cultured in Opti-MEM for 24 h. The serum-starved aortic rings were placed in a 50 μL collagen gel solution, consisting of 80% Cellmatrix Type I-A (Nitta Gelatin, Osaka, Japan), 10% 10× DMEM (Sigma-Aldrich, St. Louis, MO, USA), 0.22% NaHCO_3_, 20 mM HEPES, and 5 mM NaOH, which was pre-seeded in 96-well plates on ice. The plates were then incubated at 37 °C for 30 min. Gel-embedded aortic rings were cultured in 100 μL/well GM for 6 days and captured using an EVOS FL Auto microscope. Angiogenesis was quantified using ImageJ software version 1.52a. The ends of neovessels were joined to outline the total area. Neovascular sprouting area was calculated as the difference between the number of pixels of the total area and the aortic area defined by the circumference of the ring [28].

### 2.8. Statistical Analysis

The results were presented as the mean ± standard error. Statistical comparisons between two groups were performed using the unpaired two-tailed Student’s *t*-test after the *F* test, and between multiple groups using Scheffe’s *F* test after one-way analysis of variance. Statistical significance was set at *p* < 0.05.

## 3. Results

### 3.1. Nucleolin Localization and iSN04 Incorporation in A10 Cells

**Figure 1:**
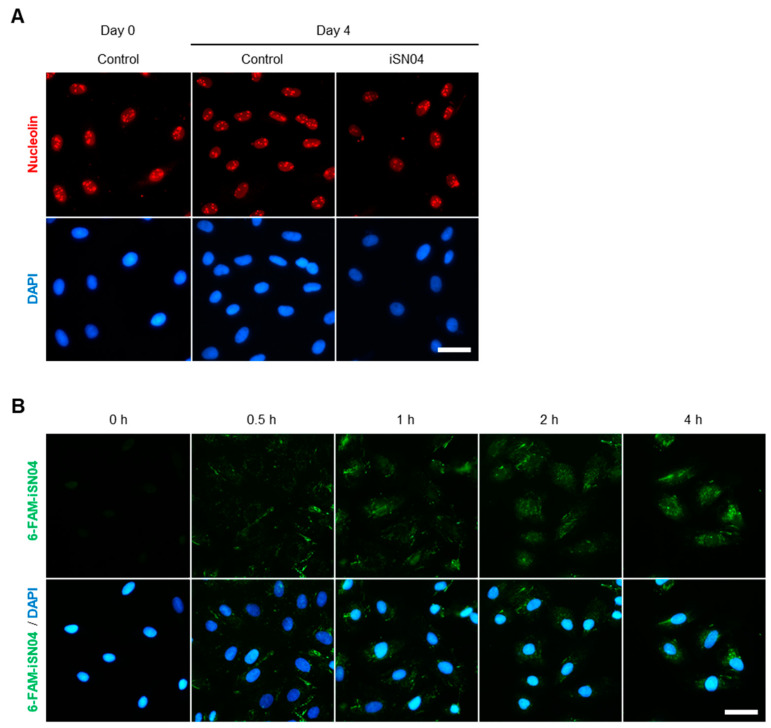
Nucleolin localization and iSN04 incorporation in A10 cells. (**A**) Representative fluorescence images of nucleolin staining of A10 cells in GM (day 0) and DM with or without 10 μM iSN04 (day 4). Scale bar, 50 μm. (**B**) Representative fluorescence images of A10 cells treated with 5 μg/mL 6-FAM-iSN04 in GM. Scale bar, 50 μm. 6-FAM, 6-carboxyfluorescein; DAPI, 4′,6-diamidino-2-phenylindole; DM, differentiation medium; GM, growth medium.

The effect of iSN04 on VSMCs was investigated using the embryonic rat thoracic aortic SMC line A10, which resembles de-differentiated SMCs and neointimal cells [29]. Immunostaining revealed that nucleolin is expressed and localized in the nuclei, particularly in the nucleoli, of proliferating A10 cells in GM (Figure 1A, day 0), as previously reported in rat aortic SMCs [18]. Nucleolin localization was not altered during differentiation induced by DM over 4 days regardless of iSN04 treatment (Figure 1A, day 4). iSN04 must be taken up into the cytoplasm and then into the nucleoplasm to reach nuclear nucleolin. Intracellular uptake of iSN04 without a carrier has been reported in myoblasts [9]. As shown in Figure 1B, 6-FAM-iSN04 was autonomously internalized into A10 cells within 30 min and diffused into the cytoplasm and nucleoplasm within 4 h, which is generally observed for oligodeoxynucleotide transport by gymnosis [9,30]. These results demonstrate that iSN04, as an anti-nucleolin aptamer, is able to act intracellularly in A10 cells.

### 3.2. iSN04 Suppresses Proliferation and Promotes Differentiation of A10 Cells

**Figure 2:**
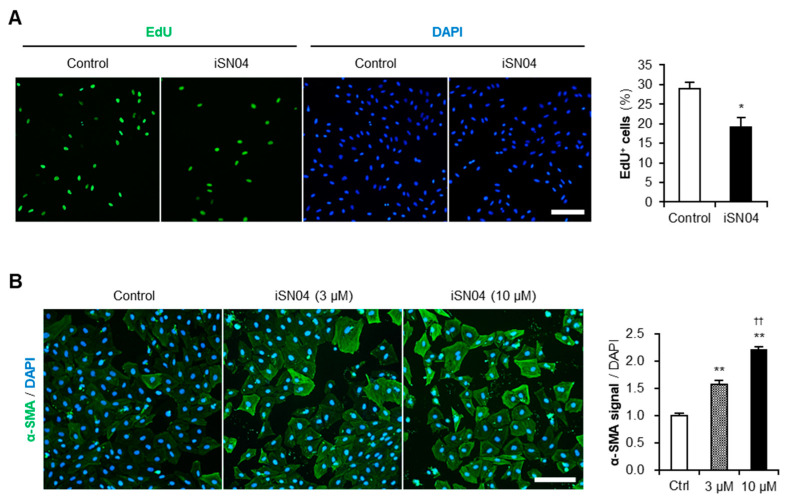
The effect of iSN04 on proliferation and differentiation of A10 cells. (**A**) Representative fluorescence images of EdU staining of A10 cells pre-treated with 30 μM iSN04 in GM for 24 h and then with 10 μM EdU in GM for 3 h. Scale bar, 200 μm. The ratio of EdU^+^ cells was quantified. * *p* < 0.05 vs. control (Student’s *t*-test). *n* = 4. (**B**) Representative fluorescence images of α-SMA staining of A10 cells treated with 3 or 10 μM iSN04 in DM for 4 days. Scale bar, 200 μm. α-SMA signal intensities per number of DAPI^+^ nuclei were quantified. ** *p* < 0.01 vs. control; ^††^ *p* < 0.01 vs. 3 μM iSN04 (Scheffe’s *F* test). *n* = 7. DAPI, 4′,6-diamidino-2-phenylindole; DM, differentiation medium; EdU, 5-ethynyl-2′-deoxyuridine; GM, growth medium; α-SMA, α-smooth muscle actin.

To verify the effect of iSN04 on proliferation, A10 cells were subjected to EdU staining. iSN04 treatment significantly decreased the ratio of EdU^+^ cells replicating genomic DNA (Figure 2A), indicating that iSN04 delayed the cell cycle. Next, the effect of iSN04 on A10 cell differentiation was quantified by α-SMA staining. As shown in Figure 2B, iSN04 significantly increased α-SMA signal intensities per cell in a dose-dependent manner during the induction of differentiation. These data demonstrate that antagonizing nucleolin with iSN04 suppresses proliferation and promotes differentiation of A10 cells.

### 3.3. iSN04 Suppresses Proliferation and Promotes Differentiation of hAoSMCs

**Figure 3:**
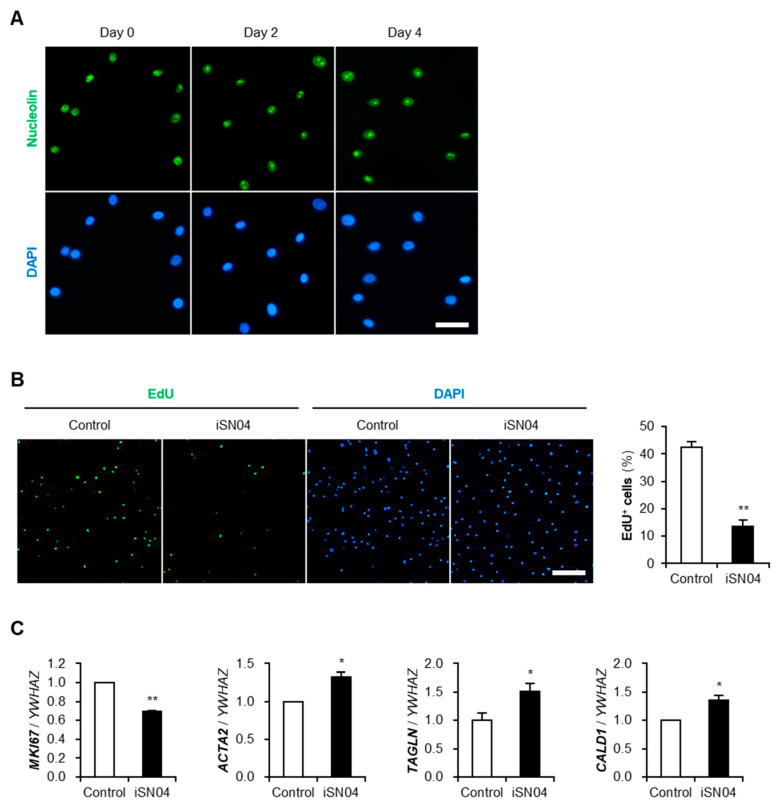
The effect of iSN04 on proliferation and differentiation of hAoSMCs. (**A**) Representative fluorescence images of nucleolin staining of hAoSMCs in hGM (day 0) and hDM (day 4). Scale bar, 50 μm. (**B**) Representative fluorescence images of EdU staining of hAoSMCs pre-treated with 30 μM iSN04 in hGM for 48 h and then with 10 μM EdU in hGM for 12 h. Scale bar, 200 μm. The ratio of EdU^+^ cells were quantified. ** *p* < 0.01 vs. control (Student’s *t*-test). *n* = 4. (**C**) qPCR results of a cell-cycle marker gene, Ki-67 (*MKI67*), and contractile SMC marker genes, α-SMA (*ACTA2*), SM22α (*TAGLN*), and caldesmon (*CALD1*), in hAoSMCs treated with 30 μM iSN04 in hGM for 48 h. * *p* < 0.05; ** *p* < 0.01 vs. control (Student’s *t*-test). *n* = 3. DAPI, 4′,6-diamidino-2-phenylindole; DM, differentiation medium; EdU, 5-ethynyl-2′-deoxyuridine; GM, growth medium; hAoSMC, human aortic smooth muscle cell; hDM, differentiation medium for human aortic smooth muscle cell; hGM, growth medium for human aortic smooth muscle cell; qPCR, quantitative real-time RT-PCR; SMC, smooth muscle cell.

To evaluate the effect of iSN04 on human VSMCs, primary cultured hAoSMC stocks were used. As shown in Figure 3A, immunostaining revealed that nucleolin is expressed and localized in the nucleoplasm and nucleoli of hAoSMCs, as observed in A10 cells in both proliferating and differentiating conditions. Since iSN04 promotes myogenesis of both murine and human myoblasts [9], it was assumed that iSN04 would not only affect rat A10 cells but also hAoSMCs. EdU staining showed that iSN04 significantly suppressed the growth of hAoSMCs (Figure 3B). Correspondingly, qPCR results indicated that iSN04 treatment significantly decreased the mRNA level of Ki-67 (*MKI67*), a cell proliferation marker that is highly expressed in the S phase (Figure 3C). This is consistent with our previous study that reported that iSN04 downregulates Ki-67 in rhabdomyosarcoma cells [12]. The expression of the contractile SMC markers α-SMA (*ACTA2*), SM22α (*TAGLN*), and caldesmon (*CALD1*) were significantly upregulated by iSN04 (Figure 3C). α-SMA is the thin filament actin that is highly expressed in differentiated VSMCs and contributes to vascular motility and contraction. SM22α is the actin-binding protein involved in Ca^2+^-independent contraction and known as an early marker of SMC differentiation. Caldesmon is the actin- and calmodulin-binding protein that regulates SMC contraction. iSN04-induced upregulation of these markers reflects the differentiation of hAoSMCs to a contractile phenotype. Altogether, these data clearly demonstrate that iSN04 suppresses proliferation and induces differentiation of human VSMCs, which may be applicable for clinical use.

### 3.4. iSN04 Suppresses Aortic Ring Angiogenesis

**Figure 4:**
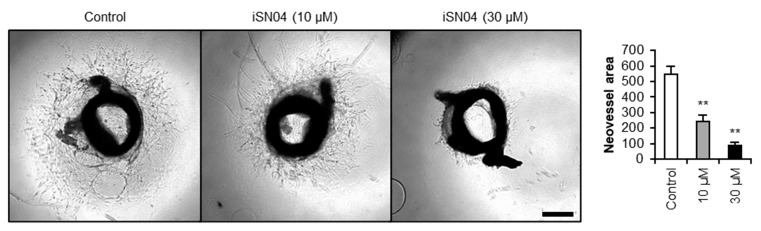
iSN04 suppresses angiogenesis of aortic rings. Representative microscopic images of iSN04-treated mouse aortic rings. Aortas from 9-week-old C57BL/6J mice were embedded in type I-A collagen gel, serum starved for 24 h, and treated with 10 or 30 μM iSN04 in GM for 6 days. Neovascular sprouting area was quantified as the difference between the total area (contiguous outline of neovascular ends) and the aortic area (circumference of the aortic ring). Scale bar, 200 μm. ** *p* < 0.01 vs. control (Scheffe’s *F* test). *n* = 5–6. GM, growth medium.

Phenotype switching of VSMCs is a mechanism of pathological angiogenesis that contributes to atherosclerotic plaque formation, growth, and rupture [5,6,7]. Since inhibition of angiogenesis renders atherosclerotic lesions small and stable, it may be a potential therapeutic strategy [7]. The aortic ring assay is a three-dimensional model of pathophysiological microvessel formation used to test the anti-angiogenic effect of molecules ex vivo [27]. As shown in Figure 4, mouse aortic rings were embedded in collagen gels and cultured with or without iSN04 for 6 days. The neovascular sprouting area was significantly reduced by iSN04 treatment, indicating that iSN04 suppresses neoangiogenesis in the aorta.

## 4. Discussion

The present study demonstrated that the 18-base myogenetic anti-nucleolin aptamer, iSN04, suppresses proliferation and promotes differentiation of VSMCs, resulting in the inhibition of aortic ring angiogenesis. Nucleolin has been reported to promote proliferation and inhibit differentiation of VSMCs, and nucleolin knockdown reverses the cellular phenotypes [19,20,21]. Our results of nucleolin antagonism by iSN04 are consistent with these findings and provide a novel biomolecule for nucleolin inhibition in VSMCs. It is known that overexpression and/or abnormal subcellular localization of nucleolin is also involved in tumor growth [31] and viral infection [32]. The 26-base guanine-rich anti-nucleolin aptamer, AS1411, has been used in phase II clinical trials for acute myeloid leukemia and renal cell carcinoma [33], which confirmed the safety and anti-proliferative effect of nucleolin inhibition by aptamers. This shows that the in vivo effect of iSN04 on VSMC de-differentiation should be tested using animal models of atherosclerosis, such as ApoE knockout mice, and neointima formation by carotid artery ligation or injury.

The mechanism of action of iSN04 in VSMCs needs to be further investigated for clinical application. The effect of iSN04 on VSMCs closely resembles its effect on myoblasts [9]. Previous studies reported that antagonism of nucleolin by iSN04 or AS1411 increased p53 protein levels in myoblasts and glioma cells, respectively [9,33], because nucleolin normally binds to the 5′ untranslated region of p53 mRNA to inhibit its translation [34,35]. During SMC differentiation, p53 directly binds to the promoter region of myocardin, a transcription factor that induces muscle-specific gene expression, to upregulate its transcription [36]. Thus, SMC-specific overexpression of p53 increases α-SMA (*ACTA2*) [36], as iSN04 did in this study (Figure 2B and Figure 3C), and suppresses injury-induced neointima formation [36]. In addition, endogenous levels of p53 protect VSMCs from apoptosis and prevent plaque formation [37]. In the plaque of p53/ApoE knockout mice, the ratio of α-SMA^+^ cells was decreased and that of Ki-67^+^ (*MKI67*) cells was increased [37], which is consistent with iSN04 reducing Ki-67 and increasing α-SMA levels (Figure 3C). These results suggest that iSN04-induced increases in p53 protein expression contribute in part to the suppression of cell proliferation, shown as a decrease in EdU^+^ cells (Figure 2A and Figure 3B), and to the induction of VSMC differentiation detected as increases in α-SMA, SM22α (*TAGLN*), and caldesmon (*CALD1*) (Figure 3C).

In addition to de-differentiation, inflammatory responses in VSMCs within the atherogenic lesion contribute to plaque initiation, progression, and rupture [38]. Therefore, anti-inflammatory treatment is also required for the prevention and therapy of atherosclerosis. Our previous study reported that iSN04 suppresses tumor necrosis factor-α (TNF-α)-induced expression of interleukin (IL)-6, IL-8, and monocyte chemoattractant protein 1 (MCP-1) in VSMCs [17]. Because nucleolin interferes with β-catenin degradation through Ser9 phosphorylation of glycogen synthase kinase 3β (GSK3β) [39], nucleolin inhibition by iSN04 reduces nuclear accumulation of β-catenin with NF-κB, resulting in suppression of inflammatory transcription [17]. β-catenin expression is also induced in VSMCs after injury to facilitate neointima formation, and β-catenin inhibitors prevent VSMC proliferation [40]. Thus, the decrease in β-catenin protein levels by iSN04 may be another mechanism for the inhibition of VSMC growth.

Furthermore, apoptosis, autophagy, and senescence of VSMCs are known to be involved in atherosclerosis [41]. Pro-inflammatory cytokines and oxidative stress promote apoptosis of VSMCs at the fibrous cap and subsequent plaque rupture. Autophagy is part of the mechanism that regulates apoptosis. For example, oxLDL disrupts autophagy in VSMCs, which fails to remove damaged mitochondria and leads to apoptosis. Defective autophagy also accelerates VSMC senescence in the plaque. Although the relationship between VSMC senescence and plaque development is not fully understood, senescent VSMCs have been implicated in fibrous cap thinning via decreased extracellular matrix and increased inflammatory responses [41]. These findings suggest that apoptosis, autophagy, and senescence of VSMCs may be therapeutic targets for prevention and treatment of atherosclerosis. The effect of iSN04 on these phenomena needs to be tested for further application.

Switching de-differentiated VSMCs to a differentiated state is a key strategy for the prevention and treatment of widespread vascular diseases such as atherosclerosis [2], neointima formation after injury and during restenosis [8], and pathological angiogenesis in atherosclerosis, arthritis, diabetic retinopathy, or cancer [7]. iSN04 is the only 18-base anti-nucleolin aptamer that can be chemically synthesized and modified on a large scale at low cost, which would be suitable for a nucleic acid drug [42]. The treatment of vascular diseases with nucleic acid aptamers has been studied. Pegaptanib (Macugen) is an anti-vascular endothelial growth factor (VEGF) 165 aptamer for neovascular age-related macular degeneration and the first therapeutic aptamer approved by the U.S. Food and Drug Administration (FDA) in 2004. However, pegaptanib is now rarely used because later anti-VEGF antibodies such as ranibizumab or soluble VEGF receptors such as aflibercept are more effective [43]. As of the end of 2023, the FDA has approved only two aptamers: pegaptanib and avacincaptad pegol (Izervay), an anti-C5 complement aptamer for geographic atrophy. The development of novel candidates is important to improve aptamer therapy in clinical settings. Several aptamers have been generated for use as therapeutic agents for cardiovascular diseases [44]. Von Willebrand factor (vWF), which causes shear-dependent thrombosis, is a mediator of atherosclerosis. Anti-vWF aptamers have been shown to block thrombus formation, which has been confirmed in clinical trials [44,45]. In addition, the anti-angiotensin II aptamers FLC112 and FLC125 were reported to inhibit proliferation of human VSMCs, and to reduce serum sodium levels and increase urine volume in Wister rats [46]. Recently, avβ3 ssDNA, an anti-integrin avβ3 aptamer, was found to attenuate proliferation and migration of rat VSMCs [47]. These outcomes demonstrate that aptamers are promising drugs for atherosclerosis. Lipid-lowering statins and anti-inflammatory agents are the current standard-of-care treatments used to stabilize and reduce atherosclerotic plaques [48]; however, some patients respond poorly to statins or have residual cardiovascular risk [49]. An alternative type of drug, such as nucleic acid aptamers, is needed. The present study demonstrated that the anti-nucleolin aptamer iSN04 may be useful for the treatment of vascular diseases. Its efficacy and safety should be further investigated in animal models in future studies.

## 5. Conclusions

An 18-base myogenic anti-nucleolin aptamer, iSN04, suppressed proliferation and promoted differentiation of VSMCs, resulting in inhibition of aortic angiogenesis. iSN04 may be a nucleic acid drug used to treat de-differentiated VSMCs in vascular diseases such as atherosclerosis, neointima formation, and pathological angiogenesis.

## Figures and Tables

**Table 1:** Primer sequences for qPCR.

Gene	Sequence (5′-3′)	Reference
*ACTA2*	ACTGCCTTGGTGTGTGACAA CACCATCACCCCCTGATGTC	[24]
*CALD1*	CTGCTCCCAAACCTTCTGAC GATTGCTTTTCCCAGAGGTTC	[25]
*MKI67*	AAGAGGTGTGCAGAAAATCCAAAG CTTCACTGTCCCTATGACTTCTGGTT	[26]
*TAGLN*	CACAAGGTGTGTGTAAGGGTG GGCTCATGCCATAGGAAGGAC	[24]
*YWHAZ*	CAAGCATACCAAGAAGCATTTGA GGGCCAGACCCAGTCTGA	[9]

## Data Availability

The data presented in this study are available on request to the corresponding author.
